# Regulatory Role of IGF2BP2 in Intestinal Mucosal Barrier Dysfunction in Ulcerative Colitis

**DOI:** 10.5152/tjg.2025.24192

**Published:** 2025-01-06

**Authors:** Ruifan Li, Bin Gu, Anli Lv

**Affiliations:** 1 Department of Pediatrics, The First Affiliated Hospital of Chengdu Medical College, Chengdu, China; 2 Department of Orthopedic, 363 Hospital, Chengdu, China; 3 Department of Ultrasound, 363 Hospital, Chengdu, China

**Keywords:** Ulcerative colitis, Intestinal mucosal barrier dysfunction, IGF2BP2

## Abstract

**Background/Aims:**

Ulcerative colitis (UC), an idiopathic and chronic inflammatory disease, primarily targets the mucosal lining of the colon. This research endeavors to reveal the mechanism of insulin-like growth factor 2 mRNA-binding protein 2 (IGF2BP2) and nuclear receptor coactivator-3 (NCOA3) in UC-induced intestinal mucosal barrier dysfunction.

**Materials and Methods:**

Dextran sodium sulfate (DSS) was used for UC mouse modeling, followed by an assessment of the disease activity index, intestinal barrier integrity, and intestinal permeability assessment through FITC-glucan assay. microRNA (miR)-222-3p, IGF2BP2, and NCOA3 levels in colon tissues of mice were detected. The targeted binding of miR-222-3p to IGF2BP2 was determined using a dual-luciferase assay. The enrichment of IGF2BP2 or N6-methyladenosine (m6A) on NCOA3 mRNA in YAMC cells was tested by RNA immunoprecipitation and m6A RNA immunoprecipitation assays, and the mRNA stability of NCOA3 was determined after actinomycin D treatment.

**Results:**

miR-222-3p was increased while IGF2BP2 and NCOA3 were decreased in the colon tissues of UC mice. IGF2BP2 overexpression effectively alleviated intestinal injury and reinstated the functional integrity of the mucosal barrier in DSS mice. IGF2BP2 recognized and bound to the m6A site of NCOA3 and increased mRNA stability, and miR-222-3p negatively regulated IGF2BP2. NCOA3 downregulation abated the beneficial impact of IGF2BP2 overexpression on DSS mice. miR-222-3p downregulation upregulated IGF2BP2/NCOA3 expression to protect against intestinal mucosal barrier dysfunction.

**Conclusion:**

IGF2BP2 was repressed by miR-222-3p, yet IGF2BP2 increased the stability of NCOA3 mRNA via an m6A-dependent pathway, ultimately leading to attenuation of UC-related intestinal mucosal barrier impairment and UC progression.

**Main Points**

- miR-222-3p targets and inhibits IGF2BP2 expression
- Overexpression of IGF2BP2 alleviates DSS-induced intestinal mucosal barrier injury in mice.
- IGF2BP2 recognizes and binds to the m6A-modified region of NCOA3 mRNA, promoting NCOA3 stability and alleviating DSS-induced intestinal mucosal barrier injury in mice.

## Introduction

Ulcerative colitis (UC) is a multifactorial, chronic, continuous, relapsing, and immune-mediated disease of the gastrointestinal tract,^1^ which affects the colon, with an estimated global prevalence of 5 million in 2023 and increasing incidence each year.^2^ According to the current etiological understanding, UC is attributed to the joint contribution of environmental and host factors and starts from the insult of pathogens on the mucosa of the gastrointestinal tract, resulting in gut barrier dysfunction and immunoinflammatory responses.^3^ The available treatments include 5-aminosalicylate, infliximab, corticosteroid, and molecular targeting therapies, however, there is no agreement on the standard treatment of UC.^4^ Adding to the plight, most UC patients fail to respond to antibodies against inflammatory cytokines like tumor necrosis factor (TNF)-α and interleukin (IL)-12/23.^5^ Therefore, there is an urgent need to explore the deep molecular etiology of UC and seek new effective therapeutic targets.

N6-methyladenosine (m6A) modification, one basic RNA epigenetic modification, is encoded by three categories of proteins, including “writers,” “erasers,” and “readers,” and exerts a significant influence in modulating cancers and various complex diseases, including inflammatory bowel disease (IBD).^6^ Insulin-like growth factor 2 (IGF2) mRNA binding proteins 2 (IGF2BP2), has been characterized as a m6A reader that cooperates with m6A writers and recognizes RNA m6A sites to influence mRNA fate and participates in the molecular landscape of human metabolic, inflammatory diseases, and cancers.^7^ IGF2BP2 is downregulated in IBD patients compared to healthy controls.^8^ Most importantly, IGF2BP2 has been documented to be downregulated in UC tissues^9^ and remit the death of intestinal epithelial cells in UC condition.^10^ Nevertheless, the impact of IGF2BP2 in UC-induced intestinal mucosal barrier injury remains to be fully elucidated and requires the support of more data.

Nuclear Receptor Coactivator-3 (NCOA3), recognized as part of the p160/steroid receptor coactivators-3 family, is known to regulate the transcriptional potency of steroid hormone receptors.^11^ The transcriptional expression of NCOA3 is intricately linked with processes involving ubiquitination and miRNA (microRNA)-mediated regulation.^12^ The engagement or interaction of m6A modification with the NCOA3 transcription has not been investigated before, and our study is the first of its kind to validate the binding between IGF2BP2 and NCOA3 through RNA immunoprecipitation assay. On a separate note, silencing of NCOA3 has been found to aggravate the injury of lipopolysaccharide (LPS)-induced Caco-2 cells.^13^ Therefore, we speculated that NCOA3 might serve as a downstream target of IGF2BP2 to play a role in intestinal mucosal barrier injury.

miRNAs, which stand as endogenously expressed non-coding RNA species, play a pivotal role in negatively modulating gene expression at the post-transcriptional phase.^14^ A previous study of our peers has demonstrated that miRNAs act as vital biomarkers and therapeutic targets for UC.^15^ In this study, we used multiple databases to predict the upstream miRNAs of IGF2BP2 and found that miR-222-3p is one of the upstream miRNAs of IGF2BP2. Most importantly, dysregulation of miR-222-3p is associated with exacerbated inflammatory responses and oxidative stress in UC condition.^16^ However, whether miR-222-3p regulates the IGF2BP2-NCOA3 axis in UC remains to be clarified.

This study endeavored to examine the function of IGF2BP2 in dextran sodium sulfate (DSS)-induced intestinal mucosal barrier damage in mice, while also exploring IGF2BP2 underlying molecular pathways and regulators, with the ultimate goal of contributing fresh scientific evidence for optimizing UC treatment strategies.

## Materials and Methods

### Animal Experiments

All animal experiments were conducted according to the protocol approved by the animal ethics committee of The First Affiliated Hospital of Chengdu Medical College (approval number: 2021291, date: March 4, 2021)and *Guidelines for the care and use of laboratory animals in biomedical research*.^17^ We selected male C57BL/6 mice (6-8 weeks old, 16-21 g) from Guangzhou Cyagen Biological Technology Co., Ltd (SYXK(Guangdong) 2020-0242) and raised them in the environment of 22-24℃ and 12 h light-dark cycle with continuous ad libitum access to regular food and water. Lasting one week of acclimatization, after which they were enrolled in the experimental procedures.

### DSS-Induced Mouse Model

Mice assigned to the sham control group were given access to sterile distilled water. For the remaining mice in experimental groups, 3% (w/v) DSS with a molecular weight ranging from 36 to 50 kDa (Article No. 0216011090, MP Biomedicals, CA, USA) were dissolved in sterile distilled water and were drunk by mice for a duration of 1 to 5 days to induce colitis symptoms. Until day 7, mice were given sterile distilled water ad libitum for drinking.^18^ On day 4, tail vein injection with lentiviruses was conducted in mice (GenePharma, Shanghai, China) (200 μL, 5 × 10^7^ TU/mL, one time every two days with a cumulative of two injections). The DSS reagent was refreshed on day 3.

The packages of overexpression vector of IGF2BP2 (pcDNA3.1 IGF2BP2, hereinafter named IGF2BP2) and the control pcDNA3.1 empty vector (named as NC), short hairpin RNA of sh-NCOA3 (sh-NCOA3) and the control (sh-NC), miR-222-3p antagomir and the control (anta-NC) and lentiviruses were all provided by GenePharma incorporate.

According to different treatments, mice were allocated to separate groups as follows (N = 16): Sham, DSS, DSS + NC, DSS + IGF2BP2, DSS + anta-NC, DSS + anta-222, DSS + IGF2BP2 + sh-NC, and DSS + IGF2BP2 + sh-NCOA3. Then, 8 mice from each group were used for fluorescein isothiocyanate (FITC)-glucan permeability assay, and the remaining 8 mice from each group underwent cardiac puncture and blood sampling after recording disease activity index (DAI) and then were euthanatized. Colon tissues were taken for histological staining and homogenate.

### Disease Activity Index (DAI)

Throughout the experimental phase, we systematically monitored and recorded the body weight, fecal consistency, and total blood volume in the feces of mice. Quantification degree of intestinal inflammation was conducted employing the DAI based on previously established criteria^19^ (Supplementary Table 1).

### Tissue Collection

After recoding DAI, euthanasia was carried out on the mice by administering an intraperitoneal dose of 200 mg/kg pentobarbital sodium. Then, the colonic lengths and weights were ascertained. A portion of colon tissues was fixed in 4% paraformaldehyde solution and the rest colon tissues were prepared into tissue homogenate for the ensuing investigative procedures.

### Hematoxylin and Eosin (H&E) Staining

After dehydration and paraffin embedding, mouse tissue blocks were sliced into 5 μm sections followed by de-paraffining and rehydration. Sections underwent histological analysis using the hematoxylin and eosin staining kit (Servicebio, Wuhan, Hubei, China). Lesions in tissue sections were observed using an optical microscope (Sigma, St. Louis, MO, USA).

### FITC-Glucan Permeability Assay

Four hours before the end of experiments, mice were treated with gastric administration of FITC-glucan at a dosage of 400 mg/kg, and blood specimens were obtained via cardiac puncture. Centrifugation at 4000 g of collected blood samples was conducted for 10 min. The serum was mixed with 100 μL volumes of phosphate-buffered saline (PBS). Measurement of intensity of fluorescence was conducted by a microscope reader, and a standard curve was plotted with an excitation wavelength of 488 nm and an emission wavelength of 525 nm, followed by measurement of sample concentration.

### Enzyme-Linked Immunosorbent Assay (ELISA)

On day 7, serum was collected from mice through cardiac puncture. After that, the levels of eotaxin chemokine, keratinocyte-derived chemokine (KC), and macrophage chemoattractant protein 1 (MCP-1) were determined by Luminex (Bio-Rad, Hercules, CA, USA) and ELISA kits (R&D Systems, Inc., Minneapolis, MN, USA).

### Cell Culture and Treatment

The YAMC mouse colonic epithelial cell line (American Type Culture Collection, ATCC, Manassas, VA, USA) was maintained in Dulbecco’s modified Eagle medium (Thermo Fisher Scientific, Waltham, MA, USA) under the environment of 37℃ and 5% CO_2_. Next, transfection of YAMC cells with aforementioned lentiviral vectors and polybrene (8 μg/mL) was performed (Sigma, St. Louis, MO, USA) and the cells were selected by puromycin.

### RNA Immunoprecipitation (RIP) Assay

YAMC cells were cultured in 10 cm plates, with each plate containing 1.2 × 10^7^ cells, and were lysed on ice with immunoprecipitation buffer (P0013J, Beyotime, Shanghai, China) enriched with a 100-fold dilution of protease inhibitor cocktail and 40 U/μL of RNase inhibitor for half an hour. Next, cell lysates were utilized for repeated pipetting and were stored at −80℃ for 5 min and were thawed on ice, followed by 10 min centrifugation at 12 000 g. Incubation of cell lysates was conducted with 5 μg antibodies against IGF2BP2 (ab128175, Abcam, Cambridge, MA, USA) or immunoglobulin G (IgG; 14678-1-AP, Proteintech, Wuhan, Hubei, China), respectively, and the incubation was continuously performed overnight at a steady temperature of 4℃. Rinsing the protein A/G magnetic beads (Bimake, China) with a solution consisting of 0.1% Tween-20 in PBS for 5 consecutive times, and then the beads were mixed with cell lysate-antibody complex, followed by rotation at 4℃ for a period of 6 h. Afterward, the RNA-protein complex was washed with elution buffer (containing 150 mM NaCl, 50 mM Tris-HCl, 5 mM EDTA, 0.5 mM DTT, 0.5% NP-40, 10% SDS, and RNase inhibitor) 5 times. The complex was processed with proteinase K at 55℃ for 1 h. The collected binding RNA was used for quantitative analysis by reverse transcription-polymerase chain reaction (RT-qPCR).

### Methylated RNA Immunoprecipitation (MeRIP)

Extraction of total RNA from YAMC cells was conducted according to the previously mentioned method^20^ and was used for m6A-IP assay. According to the protocol, the antibodies against m6A (ab151230, Abcam) and mouse IgG (ab171870, Abcam) were fixed on magnetic beads. Subsequently, the total RNA was immersed in 500 μL of a binding buffer composed of 140 mM NaCl, 50 mM Tris-HCl, 5 mM EDTA, 0.5% NP-40 and RNase inhibitor along with antibody-coupled beads, and the mixture was rotated at 4℃ for 4 h. Afterward, elution of m6A-modified mRNAs from microbeads was conducted using the elution reagent, followed by purification and RT-qPCR analysis.

### RNA Stability Assay

The 6-well plates were employed to seed YAMC cells and culture of cells was sustained for 24 hours to reach 50% confluency. After that, cells were treated with actinomycin D (5 μg/mL; Sigma) and were harvested at designated time points of 0, 3, and 6 hours. The total RNA was extracted and used for RT-qPCR analysis. The mRNA value in every group was quantified and normalized to glyceraldehyde-3-phosphate dehydrogenase (GAPDH).

### Bioinformatics

Prediction of downstream target genes of miR-222-3p were performed through miRWalk (http://mirwalk.umm.uni-heidelberg.de/), TargetScan (http://starbase.sysu.edu.cn/index.php), RNAInter (http://www.rna-society.org/rnainter/), and miRDB (https://mirdb.org/index.html). The predicted interaction site between miR-222-3p and IGF2BP2 was garnered from the TargetScan website.

### Dual-Luciferase Assay

The plasmid of IGF2BP2-wild type (WT) was generated according to the binding site of miR-222-3p and IGF2BP2 3’untranslated region (UTR), and the plasmid IGF2BP2-mutant type (MUT) was generated from the mutated binding site of miR-222-3p and IGF2BP2 3’UTR. After seeding in 96-well plates, YAMC cells were cultured to attain 70% confluency., followed by transfection with Lipofectamine 2000. The cells were subjected to co-transfection of IGF2BP2-WT/MUT plasmids along with either mimics NC (75 nM) or miR-222-3p mimics. After 48 h, cells were lysed and analyzed using a luciferase reporter gene analysis system (Promega, Madison, WI, USA).

### Reverse Transcription-Polymerase Chain Reaction (RT-qPCR)

Isolation of total RNA from colon tissues and cells was conducted employing TRIzol reagent (Invitrogen, Carlsbad, CA, USA). Next, the process of reverse transcription was employed to convert the total RNA into cDNA by cDNA first strand synthesis kit (Beyotime), and cDNA was amplified using BeyoFast™ SYBR Green qPCR Mix (Beyotime). The ABI7300 system (Applied Biosystems, Inc., Carlsbad, CA, USA) was utilized for RT-qPCR. With GAPDH or U6 as internal control,^21^ the relative expression amount was calculated according to the 2^−ΔΔCt^ method.^22^ The used primers are enlisted in Supplementary Table 2.

### Western Blot Assay

Extraction of proteins from colon tissues and YAMC cells was conducted utilizing radioimmunoprecipitation assay buffer (Saint-Bio, Shanghai, China) and were centrifuged, after which supernatant was quantified using the Biscinocholic acid assay kit (Saint-Bio). After denature and electrophoresis, proteins in gel were converted into blots on nitrocellulose membranes. The membranes were blockaded with 5% skim milk for 1 hour, after which they were subjected to overnight incubation with antibodies against NCOA3 (1:1000, AF4055, Affinity Biosciences, Jiangsu, China), IGF2BP2 (1:1000, ab128175, Abcam), and β-actin (1:5000, ab8227, Abcam). After another incubation with anti-rabbit IgG (1:5000, ab7090, Abcam) at 37℃ for 30 minutes, the gel imaging system (Invitrogen), in combination with the enhanced chemiluminescence kit (Acmec, Shanghai, China), was utilized for the visualization of protein bands.

### Statistical Analysis

All data were processed by SPSS 21.0 statistical software (IBM SPSS Corp.; Armonk, NY, USA) and GraphPad Prism 8.0 software (GraphPad Software Inc.; San Diego, CA, USA) for statistical analysis and graphing. Data were verified to follow normal distribution and homogeneous variances, as tested by normality and homogeneity of variance assays. Data between two groups were analyzed by the* t *test, and data among multiple groups were analyzed by one-way or two-way analysis of variance (ANOVA), followed by post hoc test with Tukey’s multiple comparison test. *P* values were garnered from two-sided tests. *P* < .05 was indicative of differences with statistical significance, and *P* < .01 was indicative of differences with extremely statistical significance.

## Results

### IGF2BP2 Overexpression Ameliorates Intestinal Mucosal Barrier Dysfunction in DSS Mice

**Figure 1.**
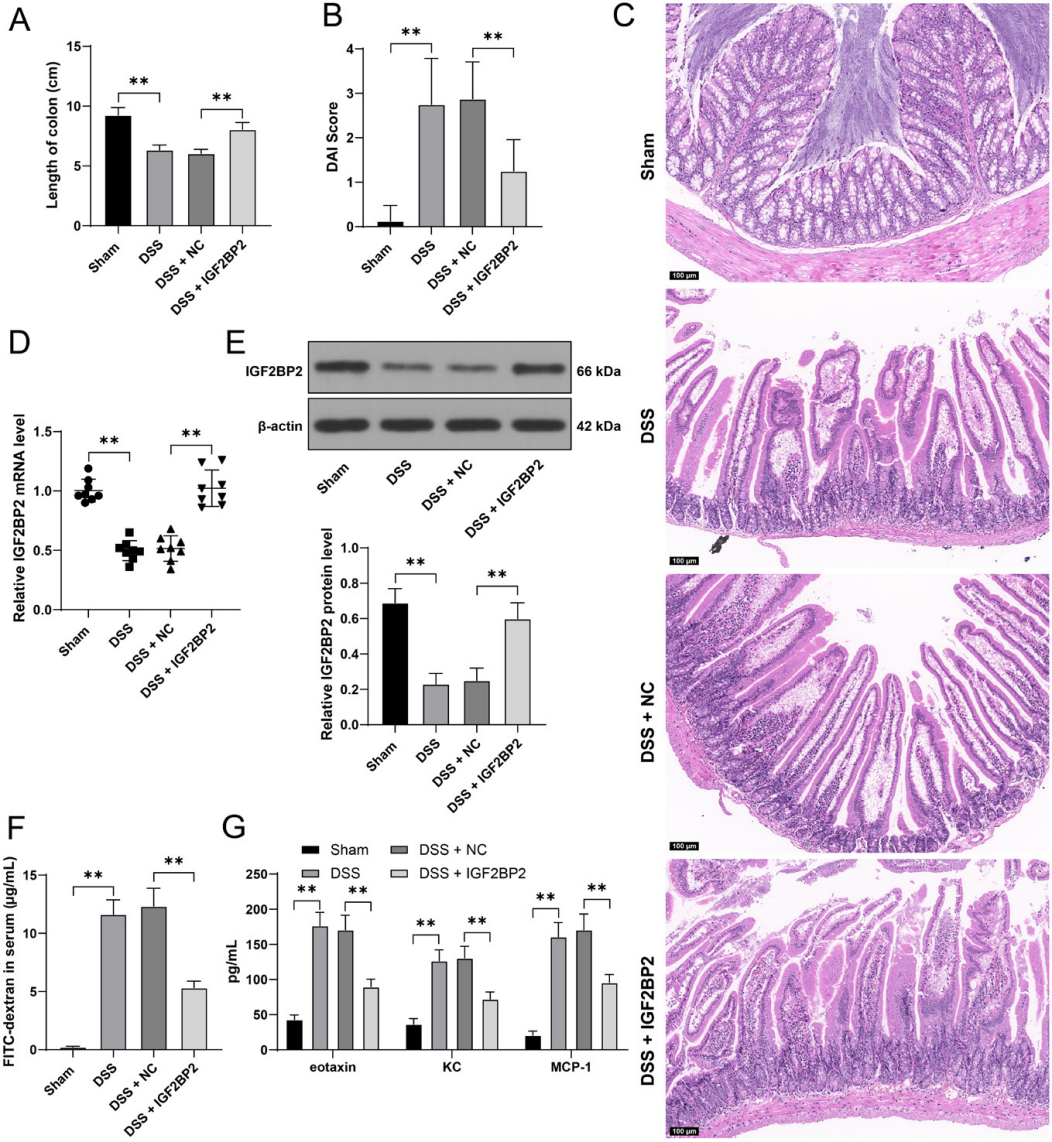
IGF2BP2 overexpression alleviates intestinal mucosal barrier injury in DSS mice. Mice were treated with DSS to induce UC and were injected with lentivirus of IGF2BP2 overexpression (IGF2BP2) via the caudal vein, with an injection of lentivirus of negative control (NC) as the control. A: Colon length of mice in different groups; B: DAI score of mice in different groups; C: H&E staining of mouse colon tissues; D-E: GF2BP2 expression levels in tissues were determined by RT-qPCR and Western blot assay; F: The concentration of FITC-labeled glucan in serum; G: The levels of eotaxin, KC, and MCP-1 in serum were determined by Luminex and ELISA. Experimental animals: N = 8, data were presented with mean ± standard deviation. Data in panels A-F were analyzed by one-way ANOVA, and data in panel G were analyzed by two-way ANOVA, followed by Tukey’s multiple comparison test. ** P < .01.

To confirm the influence of IGF2BP2 on intestinal mucosal barrier integrity in the context of DSS-induced colitis, we generated a murine model of UC by administering DSS to trigger colitis manifestations. Compared with sham mice, we found that the colons of DSS mice were shortened (*P* < .01, Figure 1A) and DAI scores of DSS mice were increased (*P* < .01, Figure 1B). Through H&E staining, we observed that compared with sham mice, DSS mice were presented with marked gastrointestinal edema and infiltration of inflammatory cells (*P* < .01, Figure 1C). The mentioned data were indicative of mouse model construction. IGF2BP2 has been documented to be decreased in UC tissues.^9,10^ Our results also revealed the downregulation of IGF2BP2 in the colon tissues of the mouse model (*P* < .01, Figure 1D and E). Subsequently, we upregulated the expression of IGF2BP2 in colon tissues of mouse models through infection of lentiviruses (*P* < .01, Figure 1D and E). Next, our observations revealed that the colon length of the mouse model was increased (*P* < .01, Figure 1A), DAI scores of DSS mice exhibited a marked decline (*P* < .01, Figure 1B), with the diminished degree of gut edema and attenuated inflammatory infiltration (Figure 1C). In addition, FITC-labeled glucan in serum was significantly increased after DSS induction and was reduced after overexpression of IGF2BP2 (*P* < .01, Figure 1F). Upon DSS administration, significant increases in the serum levels of eotaxin, KC, and MCP-1 were discerned. However, upon overexpression of IGF2BP2, these levels notably decreased (*P* < .01, Figure 1G). In light of these data, it seems that IGF2BP2 overexpression ameliorates intestinal mucosal barrier injury in DSS mice.

### IGF2BP2 Recognizes and Binds to m6A Site of NCOA3 and Promotes mRNA Stability of NCOA3

**Figure 2.**
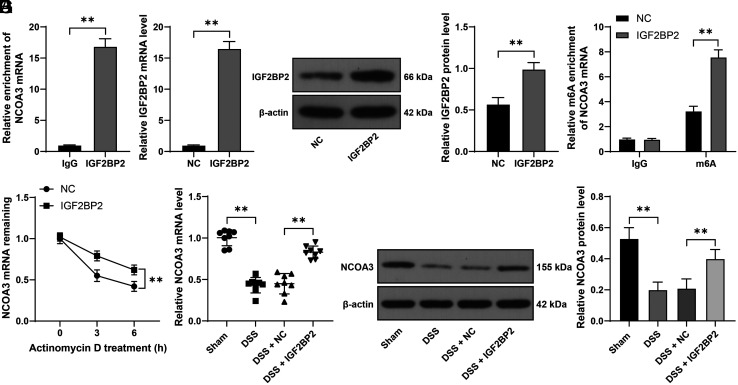
IGF2BP2 recognizes and binds to m6A site of NCOA3 and promotes mRNA stability of NCOA3. A: The binding between IGF2BP2 and NCOA3 mRNA in YAMC cells was analyzed by RIP assay; YAMC cells were infected with lentivirus of IGF2BP2 overexpression (IGF2BP2), with infection of lentivirus NC as the control; B-C: IGF2BP2 expression levels in YAMC cells were determined by RT-qPCR and Western blot assay; D: m6A modification of NCOA3 mRNA in YAMC cells was analyzed by MeRIP; E: mRNA stability of NCOA3 in YAMC cells was determined by RT-qPCR; F-G: NCOA3 expression levels in colon tissues were determined by RT-qPCR and Western blot assay. Experimental animals: N = 8, cell experiments were conducted 3 times; data were presented with mean ± standard deviation. Data in panels A-C were analyzed by the *t *test, data in panels F and G were analyzed by one-way ANOVA, and data in panels D and E were analyzed by two-way ANOVA, followed by Tukey’s multiple comparison test. ** P < 0.01.

NCOA3 is poorly expressed in LPS-induced Caco-2 cells.^13^ We speculated that NCOA3 might be the downstream mechanism of IGF2BP2. First, we conducted RIP assay in YAMC cells using an antibody against IGF2BP2, and the results showed that relative to the IgG control group, NCOA3 mRNA markedly enriched on IGF2BP2, validating the interaction between IGF2BP2 and NCOA3 mRNA (*P* < .01, Figure 2A). To validate whether NCOA3 is influenced by m6A modification and IGF2BP2 recognizes methylated NOCA3, we performed a MeRIP assay in YAMC cells. Relative to the negative control, the elevation of IGF2BP2 in YAMC cells (*P* < .01, Figure 2B and C) significantly increased the m6A level of NCOA3 (*P* < .01, Figure 2D) in YAMC cells. In addition, the mRNA stability assay showed that IGF2BP2 upregulation increased the mRNA levels of NOCA3 in YAMC cells and gradually prolonged the mRNA half-life period of NOCA3 (*P* < .01, Figure 2E). With regard to NCOA3 expression, we found that DSS induction reduced NCOA3 expression in mouse colon tissues while IGF2BP2 upregulation elevated NCOA3 expression (*P* < .01, Figure 2F and G). All in all, these observations suggested that IGF2BP2 recognizes and binds to m6A site of NCOA3 and fosters mRNA stability of NCOA3 in a m6A modification-dependent manner.

### NCOA3 Downregulation Reverses the Beneficial Impact of IGF2BP Overexpression on Intestinal Mucosal Barrier Injury in DSS Mice

**Figure 3.**
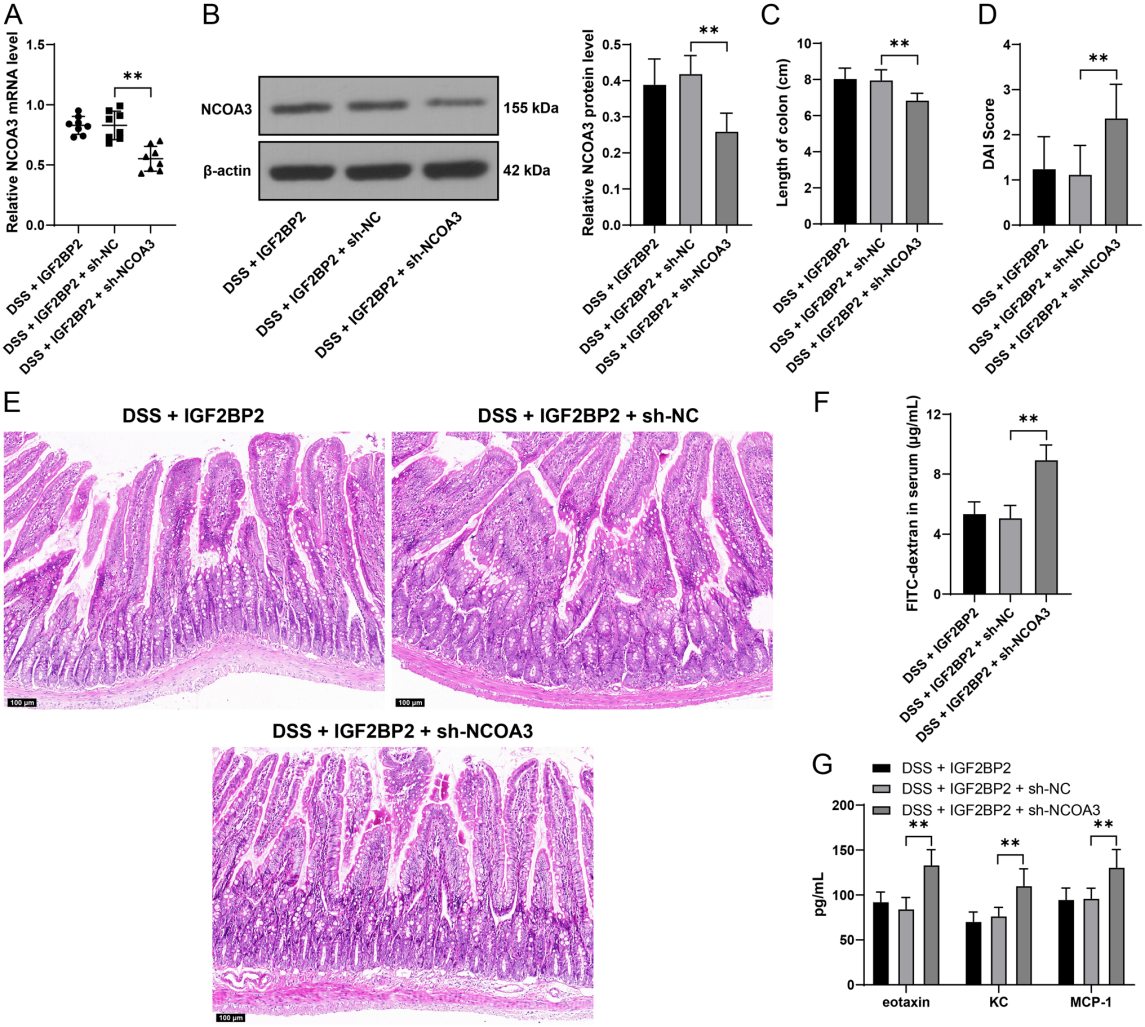
NCOA3 downregulation reverses the protective role of IGF2BP overexpression in intestinal mucosal barrier injury in DSS mice. Mice were treated with DSS to induce UC and were injected with lentivirus of NCOA3 shRNA (sh-NCOA3) via the caudal vein, with an injection of lentivirus of sh-NC as the control. A-B: NCOA3 expression levels in colon tissues of mice were determined by RT-qPCR and Western blot assay; C: Colon length of mice in different groups; D: DAI score of mice in different groups; E: H&E staining in colon tissues of mice; F: The concentration of FITC-labeled glucan in mouse serum; G: The levels of eotaxin, KC, and MCP-1 in mouse serum were determined by Luminex and ELISA. Experimental animals: N = 8, data were presented with mean ± standard deviation. Data in panels A-F were analyzed by one-way ANOVA, and data in panel G were analyzed by two-way ANOVA, followed by Tukey’s multiple comparison test. ** P < .01.

Next, we downregulated NCOA3 expression in colon tissues of experimental animals (*P* < .01, Figure 3A and B), after which the injury in DSS mice was aggravated as manifested by shortened colons (*P* < .01, Figure 3C), increased DAI scores (*P* < .01, Figure 3D), enhanced edema, and aggravated inflammatory infiltration (Figure 3E). Furthermore, upon NCOA3 downregulation, FITC-labelled glucan (*P* < .01, Figure 3F) and eotaxin, KC, and MCP-1 levels were all elevated (*P* < .01, Figure 3G). Our data concluded that NCOA3 downregulation may reverse the protective role of IGF2BP overexpression in intestinal mucosal barrier injury in DSS mice.

### miR-222-3p Targets and Inhibits IGF2BP2 Expression

**Figure 4.**
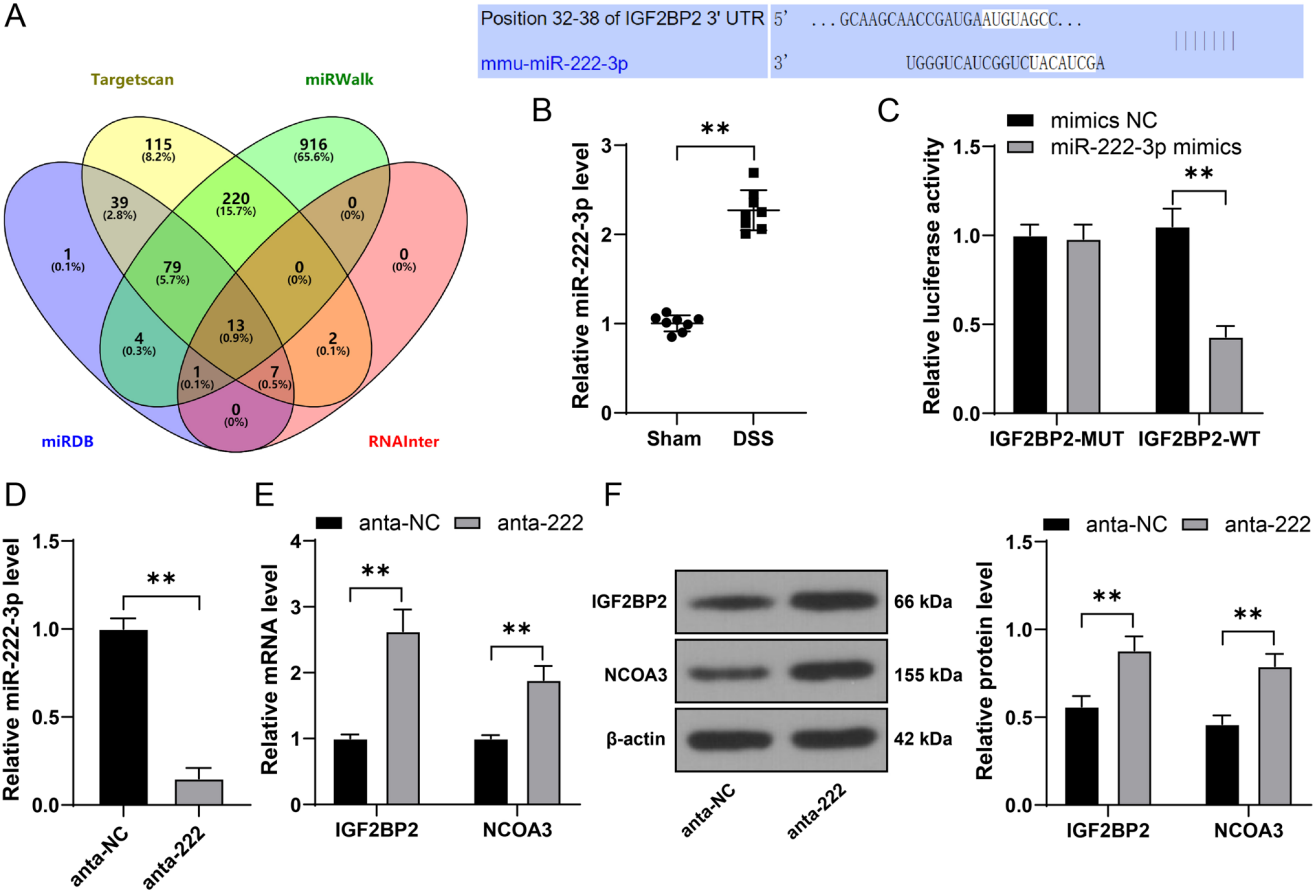
miR-222-3p targets and inhibits IGF2BP2 expression. A: The upstream miRNAs of IGF2BP2 were predicted by multiple databases and intersections of these miRNAs were garnered; B: The expression levels of miR-222-3p in colon tissues of mice were determined by RT-qPCR; C: The binding between IGF2BP2 (IGF2BP2-MUT and IGF2BP2-WT) and miR-222-3p (mimics NC and miR-222-3p mimics) in YAMC cells was analyzed by the dual-luciferase assay; YAMC cells were infected with lentivirus containing miR-222-3p antagomir (anta-222), with infection with anta-NC as the control; D: miR-222-3p expression levels in YAMC cells after infection with anta-222 or anta-NC were determined by RT-qPCR; E-F: The expression levels of IGF2BP2 and NCOA3 in YAMC cells after infection with anta-222 or anta-NC were determined by RT-qPCR and Western blot. Experimental animals: N = 8, cell experiments were conducted 3 times; data were presented with mean ± standard deviation. Data in panels B and D were analyzed by the* t *test, and data in panels C, E, and F were analyzed by two-way ANOVA, followed by Tukey’s multiple comparison test. ** P < .01.

To explore the upstream mechanism of IGF2BP2, we employed several databases to predict the upstream miRNAs of IGF2BP2 and garnered intersections of miRNAs (Figure 4A). Among these miRNAs, miR-222-3p has been reported to be upregulated in UC,^16^ and our experiment also validated the upregulation of miR-222-3p in the colon tissues of mice (*P* < .01, Figure 4B). The dual-luciferase assay in YAMC cells provided evidence supporting targeted binding between miR-222-3p and IGF2BP2 (*P* < .01, Figure 4C). We downregulated miR-222-3p expression in cells (*P* < .01, Figure 4D), after which IGF2BP2 and NCOA3 were elevated accordingly in YAMC cells (*P* < .01, Figure 4E and F). The cumulative findings implied that miR-222-3p potentially acts to suppress the IGF2BP2-NCOA3 axis.

### miR-222-3p Downregulation Alleviates Intestinal Mucosal Barrier Injury in DSS Mice

**Figure 5.**
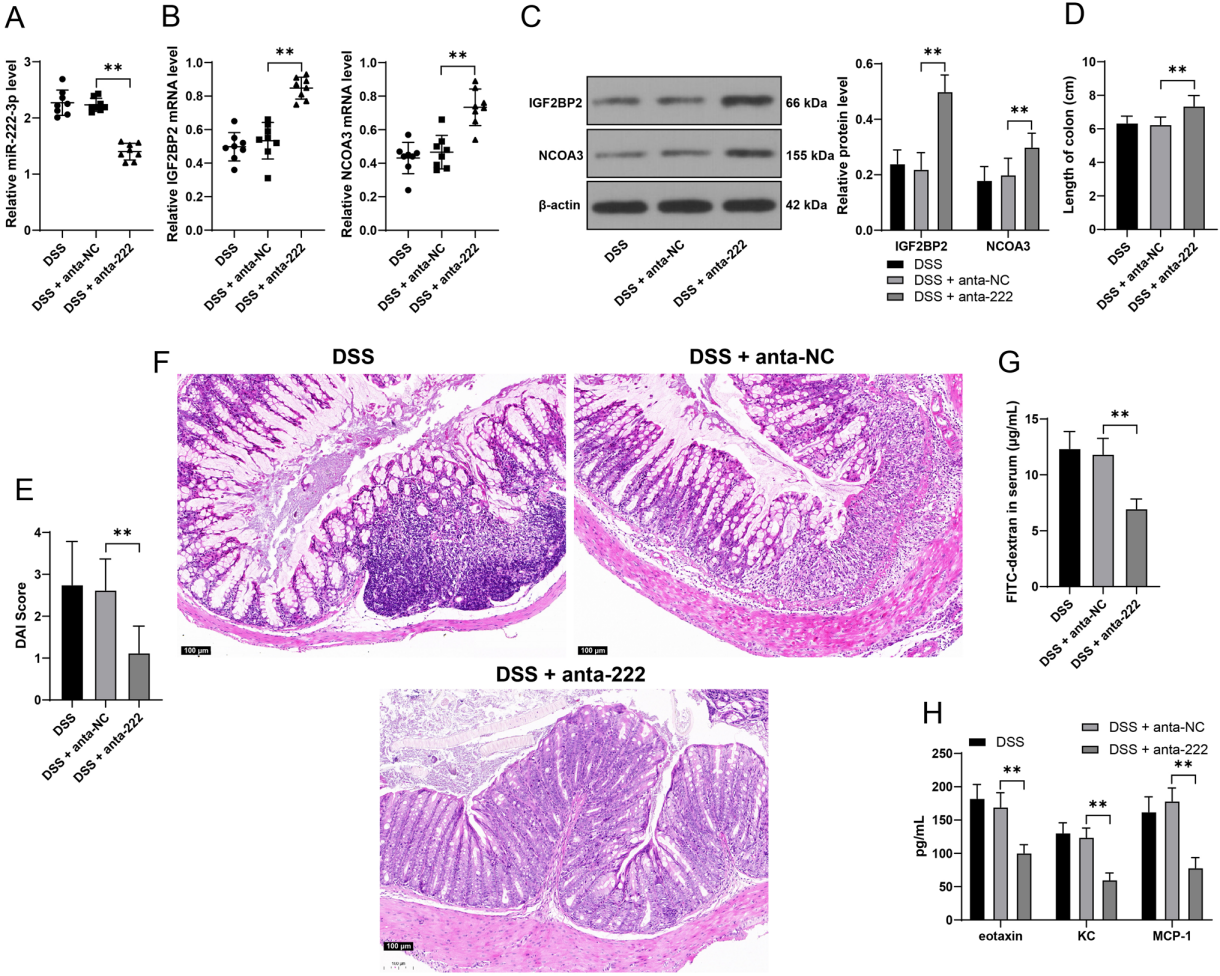
miR-222-3p downregulation alleviates intestinal mucosal barrier injury in DSS mice. Mice were treated with DSS to induce UC and were injected with lentivirus containing miR-222-3p antagomiR (anta-222) via the caudal vein, with an injection of lentivirus of anta-NC as the control. A: miR-222-3p expression levels in colon tissues of mice were determined by RT-qPCR; B-C: The expression levels of IGF2BP2 and NCOA3 in colon tissues of mice were determined by RT-qPCR and Western blot assay; D: Colon length of mice in different groups; E: DAI score of mice in different groups; F: H&E staining in colon tissues of mice; G: The concentration of FITC-labeled glucan in mouse serum; H: The levels of eotaxin, KC, and MCP-1 in mouse serum were determined by Luminex and ELISA. Experimental animals: N = 8, data were presented with mean ± standard deviation. Data in panels A-B and D-G were analyzed by one-way ANOVA, and data in panels C and H were analyzed by two-way ANOVA, followed by Tukey’s multiple comparison test. ** P < .01.

At last, we reduced miR-222-3p expression in colon tissue samples through the lentiviruses infection (*P* < .01, Figure 5A), after which the expression of IGF2BP2 and NCOA3 was increased in the colon tissues (*P* < .01, Figure 5B and C). Meanwhile, Upon miR-222-3p knockdown, the detrimental effects of DSS on mice were mitigated (*P* < .01, Figure 5D and E), and the degree of edema and inflammatory infiltration was reduced (Figure 5F). In addition, after silencing of miR-222-3p, FITC-labeled glucan in serum was reduced (*P* < .01, Figure 5G), together with reductions in eotaxin, KC, and MCP-1 (*P* < .01, Figure 5H). Altogether, miR-222-3p silencing may exert a protective role in intestinal mucosal barrier injury in DSS mice by upregulating the IGF2BP2-NCOA3 axis.

## Discussion

IBD encompasses various conditions, one of which is UC, affecting specifically the colon, and the current management of UC has been impeded by the absence of clear etiology and decisive therapeutic target.^2^ m6A modification demonstrates a pivotal role in the intestinal mucosal microenvironment and therapeutic response in UC due to its involvement in the regulation of a wide range of signaling pathways.^9^ However, there is a limited discovery of effective m6A regulators in UC. Through a spectrum of experiments, our study is the first of its kind to demonstrate that IGF2BP2 recognizes and binds to the NCOA3 m6A site to alleviate intestinal mucosal barrier dysfunction in DSS mice, and miR-222-3p negatively regulates the IGF2BP2- NCOA3 axis in DSS mice.

The breakdown of intestinal mucosal barrier is a clinical manifestation of UC and the repair of intestinal mucosa is essential for the treatment of UC.^3^ Eotaxin, KC, and MCP-1 are critical upregulated factors in UC and are correlated with inflammatory responses.^23^ With this aim, we developed a murine model of UC through DSS administration and evaluated the injury of intestinal mucosal barrier in DSS mice. We found that DSS mice were presented with severe injury of intestinal mucosal barrier as manifested by shortened colon lengths, increased DAI scores, presence of gut edema and inflammatory cell infiltration, increased intestinal permeability, and increases in eotaxin, KC, and MCP-1. Most significantly, IGF2BP2 was decreased in colon tissues of DSS mice and IGF2BP2 overexpression alleviated the dysfunction of intestinal mucosal barrier, suggesting that the downregulation of IGF2BP2 is related to the impaired intestinal mucosal barrier in UC. In support of our data, a very recent study has verified that IGF2BP2 mitigates the progression of UC by stabilizing glutathione peroxidase 4 mRNA and inhibiting intestinal epithelial cell ferroptosis.^10^ Similarly, IGF2BP2 has been also reported to switch from proinflammatory macrophage activation to anti-inflammatory activation by stabilizing tuberous sclerosis complex 1 and peroxisome proliferator-activated receptor gamma. In light of our experimental data and previous evidence, we postulated that IGF2BP2 may exert a protective function in the context of intestinal mucosal barrier dysfunction in DSS-exposed mice and it is worthwhile to explore its comprehensive upstream and downstream mechanisms.

NCOA3 acts as either a suppressor or promoter of inflammatory responses in a disease-context dependent manner. For instance, NCOA3 upregulation leads to the suppression of inflammatory responses in postoperative ileus while aggravation of inflammation in rheumatoid arthritis.^24^ Through a series of experiments, our results showed that IGF2BP2 upregulation increased the m6A levels of NCOA3 as well as the mRNA level and mRNA half-life period of NCOA3, suggesting that IGF2BP2 may recognize and bind to the m6A site of NCOA3 and then increase the mRNA stability of NCOA3. Moreover, we also found that silencing of NCOA3 enhanced the dysfunction of intestinal mucosal barrier in DSS mice in the IGF2BP2 overexpression group. In consistency with our results, NCOA3 downregulation enhances the impairment of LPS-induced Caco-2 cells* in vitro* and causes severe clinical manifestations in DSS mice *in vivo*.^13^ Based on these data, it is plausible that NCOA3 may be a downstream target of IGF2BP2 and cooperate with IGF2BP2 to protect against the injury of intestinal mucosal barrier in DSS mice.

A bunch of miRNAs have been documented to be involved in the upstream of IGF2BP2.^7^ Nevertheless, no study reports the miRNA-IGF2BP2 axis in UC. During our investigation, miR-222-3p was predicted as one of the upstream miRNAs of IGF2BP2 through multiple databases, and the direct binding between miR-222-3p and IGF2BP2 was validated by the dual-luciferase assay. A pioneering opinion implied that miR-222-3p displays elevated expression in colonic epithelial cells of UC patients and exacerbates the inflammatory responses in UC condition, while inhibition of miR-222-3p ameliorates colonic inflammation and tumorigenesis by protecting from oxidative injury in UC.^16^ miR-222-3p was upregulated in UC patients and aggravated the inflammatory response by targeting SOCS1 to activate STAT3 signaling.^25^ miR-222-3p is an important regulator of oxidative stress, the main pathogenic factor of UC.^26^ In our in vivo assays, silencing miR-222-3p alleviated the injury of intestinal mucosal barrier and increased the levels of IGF2BP2 and NCOA3 in the colon tissues of DSS mice. From the above discussion, we speculated that miR-222-3p is an inducer of the IGF2BP2-NCOA3 axis and functions as a damaging factor in the intestinal mucosal barrier integrity in DSS mice. By the way, METTL3 stimulated miR-222-3p expression by mediating the m6A modification of pri-miR-222-3p.^27^ IGF2BP2 activation reversed the METTL3 knockdown-mediated inhibition of malignancy in oral squamous cell carcinoma.^28^ There may be interactions among METTL3, miR-222-3p, and IGF2BP2, which should be illustrated in future work.

However, there are some limitations in this study. Firstly, although we validated our results in vivo, the influence of the miR-222-3p- IGF2BP2-NCOA3 axis in UC is limited to a dearth of molecular assays in cells. Secondly, the role of IGF2BP2 in the repair of intestinal mucosal barrier cannot be explained by a single mechanism, and whether the combined action of IGF2BP2 and m6A methyltransferase involved in the injury of intestinal mucosal barrier in UC requires the validation by more assays. With future endeavors, more experiments at both cell and animal levels need to be conducted to explore the deep and comprehensive mechanism of IGF2BP2, providing updated theoretical knowledge for the treatment of UC.

In summary, our findings innovatively elucidated that IGF2BP2 exhibits a protective function in the injury of intestinal mucosal barrier in DSS mice by stabilizing NCOA3 mRNA in a m6A-dependent manner, and miR-222-3p negatively regulates the IGF2BP2-NCOA3 axis in DSS mice. These results provide new theoretical knowledge for the clinical management of UC.

## Supplementary Materials

Supplementary Material

## Figures and Tables

**Supplementary Table 1.** Disease Activity Index (DAI) Scores

Weight Loss (%)	Stool Condition	Occult/Gross Bleeding	Score
None	Normal	Negative	0
1 - 5	loose stool	+	1
5 - 10	loose stool	+ +	2
10 - 20	Diarrhoea	+ + +	3
> 20	Diarrhoea	+ + + +	4

**Supplementary Table 2.** Information of PCR Primer Sequences

Gene	Sequence (5’-3’)
miR-222-3p	F: GCGCTAAGCTACATCTGGCTAC
	R: AACTGGTGTCGTGGAGTCGGC
IGF2BP2	F: ACCCTCATCACCATTTCGGC
	R: TGTTTGATGTGTGCCCCCTT
NCOA3	F: TGATGCCCCAGGCTTTCTTTA
	R: TGAGGCTGTGGTTGTGACAT
U6	F: GCTCGCTTCGGCAGCACATATA
	R: GGAACGCTTCACGAATTTGCG
GAPDH	F: GGTCCCAGCTTAGGTTCATCA
	R: AATCCGTTCACACCGACCTT

## Data Availability

The data that support the findings of this study are available on request from the corresponding author.
