# Supplementary material for: Regulatory Role of IGF2BP2 in Intestinal Mucosal Barrier Dysfunction in Ulcerative Colitis
Source: Turk J Gastroenterol. 2025 Jan 6;36(5):269–79. doi: 10.5152/tjg.2025.24192 (PMC12070433; doi:10.5152/tjg.2025.24192)
Supplement: Supplementary Material [file supplementary_material.pdf]

Supplementary Table 1. Disease Activity Index (DAI) Scores

Weight Loss (%)	Stool Condition	Occult/Gross Bleeding	Score
None	Normal	Negative	0
1 - 5	loose stool	+	1
5 - 10	loose stool	+ +	2
10 - 20	Diarrhoea	+ + +	3
> 20	Diarrhoea	+ + + +	4

Supplementary Table 2. Information of PCR Primer Sequences

Gene	Sequence (5′-3′)
miR-222-3p	F: GCGCTAAGCTACATCTGGCTAC
	R: AACTGGTGTCGTGGAGTCGGC
IGF2BP2	F: ACCCTCATCACCATTTCGGC
	R: TGTTTGATGTGTGCCCCCTT
NCOA3	F: TGATGCCCCAGGCTTTCTTTA
	R: TGAGGCTGTGGTTGTGACAT
U6	F: GCTCGCTTCGGCAGCACATATA
	R: GGAACGCTTCACGAATTTGCG
GAPDH	F: GGTCCCAGCTTAGGTTCATCA
	R: AATCCGTTCACACCGACCTT
